# Green Cataract

**Published:** 1843-03-18

**Authors:** 


#### GREEN CATARACT.

M. H. Cunier, in a report of the diseases treated at the Ophthalmic Dispensary of Brussels, invites attention to this form of cataract, which is often confounded with glaucoma, and the unfortunate patients thus abandoned to darkness. Dr. C. says, that he has met with eight cases of this kind. The individuals laboured under green cataract, which had been mistaken for incurable glaucoma. Seven of these patients were operated on and restored to sight.—*Ibid, from L'Examinateur Medical.*

